# Core Outcome Sets for Meningioma In Clinical studies (COSMIC): An international patient and healthcare professional consensus for research studies

**DOI:** 10.1093/nop/npaf023

**Published:** 2025-02-20

**Authors:** Christopher P Millward, Terri S Armstrong, Sabrina Bell, Andrew R Brodbelt, Helen Bulbeck, Linda Dirven, Paul L Grundy, Abdurrahman I Islim, Mohsen Javadpour, Sumirat M Keshwara, Shelli D Koszdin, Anthony G Marson, Michael W McDermott, Torstein R Meling, Kathy Oliver, Puneet Plaha, Matthias Preusser, Thomas Santarius, Nisaharan Srikandarajah, Martin J B Taphoorn, Carole Turner, Colin Watts, Michael Weller, Paula R Williamson, Gelareh Zadeh, Amir H Zamanipoor Najafabadi, Michael D Jenkinson

**Affiliations:** Department of Neurosurgery, The Walton Centre NHS Foundation Trust, Liverpool, UK; Institute of Systems, Molecular, & Integrative Biology, University of Liverpool, Liverpool, UK; Neuro-Oncology Branch, Center for Cancer Research, National Cancer Institute, Bethesda, Maryland, USA; The Brain Tumour Charity, Hampshire, UK; Department of Neurology, Leiden University Medical Center, Leiden, The Netherlands; Department of Neurosurgery, The Walton Centre NHS Foundation Trust, Liverpool, UK; Institute of Systems, Molecular, & Integrative Biology, University of Liverpool, Liverpool, UK; Brainstrust – The Brain Cancer People, Isle of Wight, UK; Department of Neurology, Haaglanden Medical Center, The Hague, The Netherlands; Department of Neurology, Leiden University Medical Center, Leiden, The Netherlands; Department of Neurosurgery, University Hospital Southampton, Southampton, UK; Department of Neurosurgery, The Walton Centre NHS Foundation Trust, Liverpool, UK; Institute of Systems, Molecular, & Integrative Biology, University of Liverpool, Liverpool, UK; National Centre for Neurosurgery, Beaumont Hospital, Dublin, Ireland; Department of Neurosurgery, The Walton Centre NHS Foundation Trust, Liverpool, UK; Institute of Systems, Molecular, & Integrative Biology, University of Liverpool, Liverpool, UK; Veterans Affairs Healthcare System, Palo Alto, California, USA; Department of Neurology, The Walton Centre NHS Foundation Trust, Liverpool, UK; Institute of Systems, Molecular, & Integrative Biology, University of Liverpool, Liverpool, UK; Division of Neuroscience, Florida International University, Florida, USA; Department of Neurosurgery, Copenhagen University Hospital, Copenhagen, Denmark; International Brain Tumour Alliance, Tadworth, UK; Nuffield Department of Surgical Sciences, University of Oxford, Oxford, UK; Division of Oncology, Department of Medicine, Medical University of Vienna, Vienna, Austria; Department of Neurosurgery, Addenbrooke’s Hospital & University of Cambridge, Cambridge, UK; Department of Neurosurgery, The Walton Centre NHS Foundation Trust, Liverpool, UK; Institute of Systems, Molecular, & Integrative Biology, University of Liverpool, Liverpool, UK; Department of Neurology, Haaglanden Medical Center, The Hague, The Netherlands; Department of Neurology, Leiden University Medical Center, Leiden, The Netherlands; Department of Neurosurgery, Addenbrooke’s Hospital & University of Cambridge, Cambridge, UK; Department of Cancer and Genomic Sciences, University of Birmingham, Birmingham, UK; Department of Neurology, University Hospital and University of Zurich, Zurich, Switzerland; Department of Health Data Science, University of Liverpool, Liverpool, UK; Department of Surgery, University of Toronto, Toronto, Ontario, Canada; Department of Ophthalmology, Leiden University Medical Centre, Leiden, The Netherlands; Department of Neurosurgery, The Walton Centre NHS Foundation Trust, Liverpool, UK; Institute of Systems, Molecular, & Integrative Biology, University of Liverpool, Liverpool, UK

**Keywords:** clinical trial, COMET, core outcome set, meningioma, outcomes

## Abstract

**Background:**

Core Outcome Sets (COS) define the minimum outcomes that should be measured and reported in all clinical trials for a specific health condition or health area. The aim was to develop 2 COS for intracranial meningioma to be used in future clinical studies: COSMIC: Intervention for effectiveness trials and COSMIC: Observation for studies of incidental/untreated meningioma.

**Methods:**

A study advisory group was formed with representation from international stakeholder groups: EORTC BTG, ICOM, EANO, SNO, RANO-PRO, BNOS, SBNS, BIMS, TBTC, International Brain Tumour Alliance, and Brainstrust. Outcomes of potential relevance to key stakeholders were identified and rationalized to populate 2 eDelphi surveys. Participants were recruited internationally and asked to rate each outcome on its importance for inclusion in the COS. The 2 final COS were ratified through 2, one-day, online consensus meetings.

**Results:**

The COSMIC: Intervention eDelphi survey contained 25 items and was completed by 199 participants. Following the consensus meeting, 15 outcomes were included. The COSMIC: Observation eDelphi survey contained 17 items and was completed by 129 participants. Sixteen outcomes were included. Eight core outcomes were common to both COS; tumor growth, physical, emotional, and neurocognitive functioning, overall quality of life, progression-free survival, meningioma-specific mortality and overall survival. Role and social functioning were core outcomes in COSMIC: Observation but not COSMIC: Intervention.

**Conclusions:**

Uptake of these COS in relevant future meningioma clinical studies will ensure that stakeholder-determined, critically important outcomes are consistently measured and reported across similar clinical studies.

**Key points**

- Meningioma outcome measurement is heterogeneous and requires standardization.
- Two Core Outcome Sets have been developed for use in future meningioma clinical studies.
- The Core Outcome Sets currently define *what*, but not *how* each outcome should be measured.

**Importance of the Study**

The outcomes measured in meningioma clinical trials are not standardized. Until now, no initiative had asked what outcomes matter most to key stakeholders (including meningioma patients), and what outcomes should always be measured, as a minimum, in meningioma clinical trials. We have developed 2 Core Outcome Sets (COS) that define what outcomes should be measured and reported, as a minimum in future meningioma clinical effectiveness trials (COSMIC: Intervention) and clinical studies of incidental/untreated meningioma (COSMIC: Observation). This was achieved by recruiting international participants to 2 eDelphi surveys, prepopulated with outcomes of potential relevance to key stakeholders. Each COS was ratified following an online consensus meeting. The COS should be used in future meningioma clinical studies to ensure stakeholder-determined, critically important outcomes are consistently measured and reported, allowing for comparison across similar studies. Future work will determine how each outcome should be measured.

Most meningioma clinical trials have investigated treatment options for patients with high-grade, recurrent, and progressive disease.^1-5^ There is considerable interest in performing high-quality clinical trials to optimize management strategies for patients at the start of the treatment pathway (eg, the role of adjuvant radiotherapy after resection of atypical meningioma),^3^ for those who may be poor surgical candidates, have inoperable, residual, or recurrent disease, or after conventional options have been exhausted. However, the outcomes measured in meningioma clinical trials are not standardized. This was demonstrated in part by a comprehensive review, which aimed to identify historical outcomes measured in trials of systemic therapies in order to establish endpoint benchmarks for future clinical trials of medical therapies for recurrent intracranial meningioma.^6^ There was marked heterogeneity in the definition of response criteria and the measured survival outcomes; for instance, some studies reported median overall survival, whilst others reported median progression-free survival. Only progression-free survival at 6 months (PFS-6) was found to be common to all but one of the studies evaluated. Consequently, PFS-6 has been proposed as the outcome to measure in future studies evaluating interventions for those who have progressed after local therapies have failed.^6^

The Response Assessment in Neuro-Oncology (RANO) Meningioma Working Group published recommendations for assessing response and progression in clinical trials involving patients with meningioma, due to lack of consensus on optimal endpoints and variation in trial design and response criteria.^7^ Whilst both of these initiatives highlight concerns regarding outcome measurement and reporting heterogeneity for specific issues (namely response and progression), no initiative has asked what outcomes matter most to key stakeholders (including meningioma patients) and what outcomes should always be measured, as a minimum, in meningioma clinical studies.

Clinical studies of incidental and untreated intracranial meningioma are uncommon, mostly observational, single-center, and retrospective in design.^8^ International consensus guidelines currently recommend interval MRI monitoring as the first-line management strategy for an incidental meningioma, but the interval timing, follow-up duration, and treatment indications are not defined.^9^ There is heterogeneity in imaging frequency and variation in clinical management between active long-term MRI and clinical monitoring or upfront surgery or radiotherapy/radiosurgery.^10^ The balance of the risks and benefits of active surveillance versus upfront treatment is also not well defined.^11^

Recent work has attempted to accurately define risk factors for untreated meningioma growth. The Asan Intracranial Meningioma Scoring System (AIMSS) and Incidental Meningioma: Prognostic Analysis Using Patient Comorbidity and MRI Tests (IMPACT) calculator stratify patients based on the clinical and imaging features into risk groups.^12,13^ Definitions of growth or progression have not been uniform, which has hampered the synthesis of results; for instance, some studies report absolute changes in tumor size, whilst others report relative changes in tumor size. The RANO Meningioma Working Group has recommended that change with relation to time (rate) should be used, but this has been inconsistently applied.^7^

A Core Outcome Set (COS) is defined as the *minimum* set of outcomes that should be measured and reported in all clinical trials for a specific health condition or health area.^14^ COS development is in its infancy within the field of neuro-oncology, but efforts are underway for pediatric brain tumors and have recently been completed for glioma.^15,16^

COS development and uptake for meningioma would ensure that the outcomes that are of critical importance to key stakeholders are measured in future meningioma clinical trials and studies, and in doing so, tackle the issues described. Here, we present the results of The COSMIC Project: an international, multi-stakeholder, consensus-driven effort to develop two COS for meningioma.^17^ The results are reported in accordance with the Core Outcome Set Standards for Reporting (COS-STAR) statement.^18^

## Methods

### Study Overview

**Figure 1.**
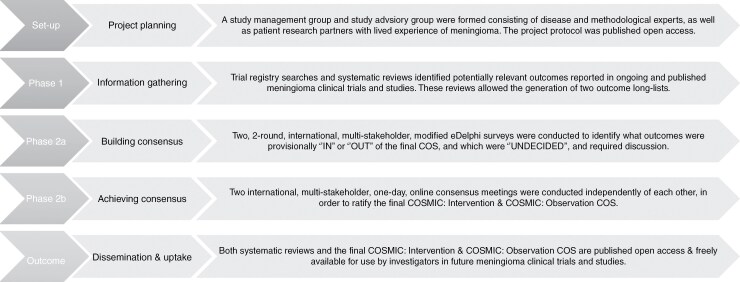
A Flow Chart Summarizing the Stages of The COSMIC Project

A study management group (SMG) was formed that consisted of meningioma and methodological experts. An internationally representative, multistakeholder study advisory group (SAG) was created, which included additional meningioma and methodological experts, academic and charitable organization representatives, and patient research partners (PRP). The study protocol was written with input from the SMG, SAG, and PRPs and contains a comprehensive description of the project methodology.^17^ The scope of the COS, project registration details, and a brief description of Phase 1 (Information gathering) and Phase 2 (Consensus building) are described below (see Figure 1).

### Scope of the COS

The scope of both COS was defined according to the Core Outcome Set Standards for Development (COS-STAD) recommendations in the published study protocol.^17,19^ In summary, COSMIC: Intervention was developed for use in interventional intracranial meningioma clinical trials that are designed to inform clinical decision-making and improve clinical care for patients. COSMIC: Observation was developed for use in observational clinical studies concerned with incidental, minimally symptomatic, and/or untreated cohorts of patients with intracranial meningioma that are designed to inform monitoring and decision to treat strategies.

### Registration

The study was registered with the Core Outcome Measures in Effectiveness Trials (COMET) database as study 1508 and accessible at https://www.comet-initiative.org/Studies/Details/1508. Institutional review board sponsorship and ethical approval were obtained (University of Liverpool, UoL001601).

## Phase 1—Information Gathering

Two systematic reviews were conducted (which also included searches of trial registries) to identify outcomes measured and reported in intracranial meningioma clinical trials and studies. The inclusion and exclusion criteria, information sources and search strategies, selection processes, data item and data collection processes, and synthesis methods have been published both *a priori* in The COSMIC Project protocol,^17^ and within the associated systematic review manuscripts for both COSMIC: Intervention^20^ and COSMIC: Observation.^21^ Our definition of a trial or study outcome and our method of data extraction are aligned with previous methods.^22^ Outcomes were classified according to the outcome taxonomy proposed by COMET.^14,23^

Data generated from each systematic review were analyzed separately to maintain 2 distinct long-lists of unique outcomes. Exact matching outcomes were deduplicated and those that remained were grouped and deduplicated further when similarities in spelling, meaning, or context existed. A working “lay” definition was provided for each outcome. The initial long lists were created by 3 members of the SMG with knowledge of the disease area and COMET methodology (CPM, AII, and MDJ). Following this, additional review and refinement were performed by 2 expert methodologists from the SMG (AGM and PRW) to ensure that (1) the long lists were appropriately rationalized for use in an eDelphi survey, and (2) contained outcomes that were rateable, but also meaningful, if included in 1 of the 2 COS. Prior to advancing both long lists to Phase 2, a final review was undertaken by SAG members, including PRPs and lay representatives.

## Phase 2—Consensus Building

### eDelphi Surveys

#### Key stakeholder eligibility criteria

1. Meningioma patients who had:

(i) Completed or received treatment for an intracranial meningioma with surgery, radiotherapy, stereotactic radiosurgery, or pharmacotherapy, either in isolation or in combination (COSMIC: Intervention eDelphi survey only).
(ii) Not received treatment for a radiologically diagnosed intracranial meningioma (COSMIC: Observation eDelphi survey only).

2. Healthcare professionals and researchers
  A member of a clinical team directly responsible for the care of patients with a meningioma (who may also be a researcher), or a researcher who may use the COS in the future (but may not have a direct clinical responsibility). (Eligible for both eDelphi surveys).
3. Caring or supporting roles

Individuals who provided a regular and involved caring or supporting role to a patient with a meningioma. (One or both eDelphi surveys dependent upon the individual participant’s self-declared experience).

#### Key stakeholder sampling and recruitment

The first eDelphi survey panel was formed of healthcare professionals and researchers (Panel 1). The second panel was formed by amalgamating the patient and caring/supporting role stakeholder groups (Panel 2). A minimum of 20 participants was needed for each panel.

1. Healthcare professionals and researchers

These were recruited nationally and internationally via personal contacts, the SAG membership, and national and international professional societies. Professional societies, groups and organizations included the British-Irish Meningioma Society (BIMS), the British Neuro-Oncology Society (BNOS), the Society of British Neurological Surgeons (SBNS), the British Skull Base Society (BSBS), the European Organization for Research and Treatment of Cancer Brain Tumour Group (EORTC BTG), the European Association of Neuro-Oncology (EANO), the International Consortium on Meningioma (ICOM), the Response Assessment in Neuro-Oncology Patient-Reported Outcome Group (RANO-PRO), and the Society for Neuro-Oncology (SNO).

2. Patients and those in caring/supporting roles

Patients and those in caring or supporting roles were approached and invited to participate via charities, support groups, and social media platforms/forums. A named contact was identified who was responsible for distributing a participant invitation email and/or social media post containing a link to the study website (thecosmicproject.org) and the online DelphiManager platform. The following named charities contributed: The International Brain Tumour Alliance (IBTA), The Brain Tumour Charity (TBTC), Brainstrust—the brain cancer people, and the Brain Tumour Foundation of Canada. Permission was granted from administrators of Facebook support groups to join and post recruitment material directly within the group, and a study 𝕏 account (formerly Twitter) was created to increase study awareness and provide direct links to the study website and DelphiManager platform.

#### Registration and consent for eDelphi participation

Individuals were directed to the study website which contained all necessary information about participation. Registration was only possible via a link provided on our website to the online DelphiManager platform. During registration, screening questions were used to establish which stakeholder group a participant belonged to, followed by additional questions to establish their eligibility for the study. Additional information was collected about participants, specific to the stakeholder group to which they belonged. eConsent was obtained for all participants, including an option to be listed as a named individual within a collaborative authorship group.

#### eDelphi survey structure and administration

Both were constructed and delivered through the online DelphiManager platform and were piloted with members of the SAG (including PRPs). Each survey consisted of 2 rounds. Round 1 for each was populated with the long-list developed in Phase 1 and offered the opportunity for participants to suggest outcomes for inclusion in Round 2. Those participants eligible to participate in both surveys did so consecutively, followed by both Round 2 surveys once available. Participant-level data was pseudo-anonymized.

#### Outcome scoring

During Round 1, participants rated the importance of each outcome presented, using a 9-point Likert scale, whereby a score in the range of (1-3) was of limited importance, (4-6) was important but not critical, and (7-9) was critically important. All outcomes from Round 1 were carried forward to Round 2 for participants to rate again. During presentation of outcomes for rating in Round 2, the Round 1 aggregate ratings for each outcome were presented as histograms demonstrating frequency of rating for each point on the Likert scale. A separate histogram was created to summarize ratings for each panel, and both were displayed to all participants in Round 2.

#### Outcome analysis

To include an outcome (consensus-in) beyond Round 2 of the eDelphi surveys, 80% or more of participants were required to score an outcome as critical (7-9) in both panels. Outcomes that were rated as critical by 50% or less of participants from both panels were dropped (consensus-out). Outcomes that were neither included nor dropped after the eDelphi surveys were advanced to the consensus meetings.

### Consensus Meetings

#### Key stakeholder eligibility criteria, sampling, and recruitment

Eligibility required participants to have completed both rounds of the associated eDelphi survey. Purposive sampling was used to select eligible eDelphi participants from each of the 2 panels from both surveys. Healthcare professionals were sampled across job roles, followed by geographical location and gender. Patients and those in supporting roles were balanced across treatment (for COSMIC: Intervention), or active monitoring experiences (for COSMIC: Observation). When the pool of eligible patient participants was large enough, an attempt was made to balance recruitment across geographical location and gender also.

#### Registration and consent for consensus meeting participation

Individuals were approached via email and invited to attend an online consensus meeting. A participant information leaflet was provided. If unavailable or unwilling to participate, further invitations were sent to new individuals. eConsent was obtained for all participants.

#### Consensus meeting structure and administration

The consensus meetings were delivered online (using the Zoom platform), independently, on consecutive days. The results of the second round of each eDelphi survey were used to determine what outcomes were dropped and what outcomes were included in the final COS, and importantly, which outcomes required discussion at a consensus meeting. The general structure of both meetings was as follows: a brief presentation to reaffirm the purpose of and expectations for the meeting, review of outcomes categorized as “consensus-in” and “consensus-out,” and presentation, discussion, and voting on outcomes that were neither “consensus-in” or “consensus-out.” Questions were presented to consensus meeting participants and anonymous electronic voting took place.

#### Outcome scoring

Following each discussion, participants were required to vote anonymously when presented with a question using the 9-point Likert scale as previously described.

#### Outcome analysis

After voting, data analysis was performed immediately (as per the eDelphi surveys) and presented back to all meeting participants. If the voting process resulted in “consensus-in” or “consensus-out,” the decision was accepted. If neither “consensus-in” or “consensus-out” was attained, further discussion and voting took place until a decision was reached.

## Results

### Phase 1—Creating Outcome Long-lists for Population of eDelphi Surveys

Our 2 independent and comprehensive systematic reviews identified outcomes of potential relevance to key stakeholders recruited into Phase 2 of this work. The long-lists were large and excessively granular. An iterative process was required to create 2 eDelphi-friendly long-lists that would be (1) not overly burdensome to participants due to length/time taken to complete, (2) composed of unambiguous, rateable outcomes with clear application to future meningioma clinical trials and studies, and (3) respectful of the rigorous methodological process undertaken prior to this point. Summaries of the conversion process from systematic review long-list to eDelphi survey long-list are provided in Supplementary Appendices 1 and 2. The final survey items are provided in Supplementary Appendices 3 and 4. The SMG and SAG elected to add emotional, social, and role functioning to the COSMIC: Intervention eDelphi survey, with the latter 2 also being added to the COSMIC: Observation eDelphi survey.

### Phase 2a—Consensus Building with eDelphi Surveys

The eDelphi surveys for COSMIC: Intervention and COSMIC: Observation were open for Round 1 between 27 May 2022 and 6 July 2022, and for Round 2 between 11 July 2022 and 28 August 2022.

### COSMIC: Intervention eDelphi Survey

#### Participant registration, characteristics, and completion data

Two hundred and fifty-two participants registered to take part: 147 within Panel 1, and 105 within Panel 2. Fifty-one percent of Panel 1 participants identified primarily as a member of a clinical team and 41% identified primarily as a meningioma researcher. Global representation was achieved with participants from the United Kingdom (52%), United States (15%), Germany (9%), and Canada (5%), amongst others. A broad spectrum of job roles were represented including neurosurgeons (66%), oncologists (11%), researchers (7%), neurologists (6%), neuropathologists (3%), neuroradiologists (3%), and specialist nurses (1%).

Nearly all Panel 2 participants were patients who had undergone treatment (96%). Three carers/family members, and 1 charity/support group representative also registered to participate. Global representation was achieved with participants from the United Kingdom (48%), United States (31%), Australia (14%), Canada (2%), and Ireland (2%), amongst others. A broad spectrum of treatment histories was identified in those registering as a patient but primarily consisted of a single surgery (57.4%), followed by surgery and radiotherapy (14.9%), as well as various other combinations.

Of the 147 Panel 1 registrants, 98% completed Round 1 and 91% completed Round 2 (7.6% attrition rate between rounds). Of the 105 Panel 2 registrants, 82% completed Round 1 and 64% completed Round 2 (22.1% attrition rate between rounds). A summary of these data is provided in Table 1.

**Table 1.** COSMIC: Intervention Registration and Survey Completion Data Combined with Panels 1 and 2 Participant Characteristic Data

Panel 1—Healthcare professionals & researchers	No. (%)	Panel 2—Patients and those in caring/supporting roles	No. (%)
			
**Registration and completion**		**Registration and completion**	
Registered	147	Registered	105
Completed Round 1	144 (98.0)	Completed Round 1	86 (82.0)
Completed Round 2	133 (90.5)	Completed Round 2	67 (63.8)
R1 to R2 attrition No. (% of those completing Round 1)	11 (7.6)	R1 to R2 attrition No. (% of those completing Round 1)	19 (22.1)
			
**Role/s within this stakeholder group**		**Role/s within this stakeholder group**	
Member of clinical team directly responsible for meningioma patients	75 (51.0)	Patient with a meningioma who has undergone treatment	101 (96.2)
Researcher involved in meningioma research	60 (40.8)	Carer/family member to meningioma patient (treated)	3 (2.6)
Both a member of a clinical team and a researcher as defined above	12 (8.2)	Charity/support group representative to meningioma patients (treated)	1 (1.0)
			
**Years in meningioma relevant practice/research**		**No. years with diagnosis or time spent in caring/supporting role**	
		Less than 2	38 (36.2)
Less than 5	30 (20.4)	2 to 4	34 (32.4)
5 to 9	33 (22.4)	5 to 9	20 (19.0)
10 to 14	32 (21.8)	10 to 14	6 (5.7)
15 to 20	27 (18.4)	15 to 19	6 (5.7)
20 or more	25 (17.0)	20 or more	1 (1.0)
			
**Gender**		**Gender**	
Male	119 (81.0)	Female	94 (89.5)
Female	26 (17.7)	Male	11 (10.5)
Prefer not to say	2 (1.4)	Prefer not to say	0 (0.0)
			
**Age**		**Age**	
18-24	6 (4.1)	18-24	0 (0.0)
25-29	11 (7.5)	25-29	0 (0.0)
30-34	12 (8.2)	30-34	3 (2.9)
35-39	20 (13.6)	35-39	5 (4.8)
40-44	25 (17.0)	40-44	7 (6.7)
45-49	27 (18.4)	45-49	19 (18.1)
50-54	23 (15.6)	50-54	16 (15.2)
55-59	17 (11.6)	55-59	19 (18.1)
60-64	4 (2.7)	60-64	18 (17.1)
65-69	1 (0.7)	65-69	11 (10.5)
70-74	1 (0.7)	70-74	5 (4.8)
75-79	0 (0.0)	75-79	2 (1.9)
			
**Country of residence**		**Country of residence**	
United Kingdom	76 (51.7)	United Kingdom	50 (47.6)
United States	22 (15.0)	United States	32 (30.5)
Germany	13 (8.8)	Australia	15 (14.3)
Canada	7 (4.8)	Canada	2 (1.9)
Switzerland	4 (2.7)	Ireland	2 (1.9)
India	4 (2.7)	Other (frequency less than 2)	4 (3.8)
Netherlands	3 (2.0)		
Spain	2 (1.4)		
Other (frequency less than 2)	16 (10.9)		
			
**Primary job role**		**Treatment/s received to date (% of *n* = 101)**	
Neurosurgeon	97 (66.0)	Surgery	58 (57.4)
Oncologist (Clinical/Medical/Radiation)	16 (10.9)	Surgery and radiotherapy	15 (14.9)
Researcher	10 (6.8)	Radiosurgery	7 (6.9)
Neurologist	9 (6.1)	Multiple surgeries and radiotherapy	6 (6.0)
Neuropathologist	5 (3.4)	Surgery and radiosurgery	5 (5.0)
Neuroradiologist	5 (3.4)	Multiple surgeries	4 (4.0)
Other	3 (2.0)	Radiotherapy	4 (4.0)
Neuro-oncology/Skull base specialist nurse	2 (1.4)	Surgery, radiotherapy, and radiosurgery	1 (1.0)
		Surgery, radiotherapy, and pharmacotherapy	1 (1.0)
		**Years since last treatment (% of *n* = 101)**	
		Less than 2	65 (64.4)
		2 to 4	24 (23.8)
		5 to 9	11 (10.9)
		10 to 14	2 (2.0)
		15 to 19	1 (1.0)

### COSMIC Intervention eDelphi Results Summary

After Round 1, the percentage of voting responses that were 7-9 for each outcome was calculated. Within Panel 1, 11 of the 24 outcomes presented had at least 80% of voting responses rated 7-9. Within Panel 2, this was 17 of 24 outcomes presented. A summary of these data is provided in Supplementary Appendix 5.

Sixty-six additional outcomes were proposed for addition to Round 2 by participants. These were reviewed by 3 members of the SMG (CPM, AII, and MDJ), which were then presented and discussed with 2 expert methodologists from the SMG (AGM and PRW), and it was agreed that only 2 should be added to Round 2. As both terms were synonymous, a single outcome was added “seizures.” A summary of outcomes proposed for addition and reasons for not adding all but 2 of them to Round 2 is provided in Supplementary Appendix 7.

Following Round 2, the percentage of voting responses that were 7-9 for each outcome was again calculated. Within Panel 1, 14 of the 25 outcomes presented had at least 80% of voting responses rated 7-9. Within Panel 2, this was 18 of 25 outcomes presented. Fourteen outcomes met the definition for inclusion in the COSMIC: Intervention COS at this stage. Three outcomes met the definition for exclusion at this stage. Eight outcomes remained that did not meet either definition. Therefore, these outcomes were progressed to the COSMIC: Intervention consensus meeting. A summary of these data is provided in Supplementary Appendix 5.

### COSMIC: Observation eDelphi Survey

#### Participant registration, characteristics, and completion data

One hundred and forty-eight participants registered: 116 within Panel 1, and 32 within Panel 2. Fifty-two percent of Panel 1 participants identified primarily as a member of a clinical team directly responsible for the care of meningioma patients, while 8% identified primarily as a researcher involved in meningioma research. Global representation was achieved with participants from the United Kingdom (54%), United States (12%), Germany (9%), and Canada (3%) amongst others. A broad spectrum of job roles were represented including neurosurgeons (69%), oncologists (9%), researchers (7%), neurologists (7%), neuroradiologists (3%), specialist nurses (3%), and neuropathologists (1%).

Most Panel 2 participants were patients with a meningioma who had not undergone treatment (90%). However, 1 carer/family member, and 2 charity/support group representatives also registered to participate. Global representation was achieved with participants from the United Kingdom (63%), United States (28%), Australia (6%), and Canada (3%). Eighty-six percent of patients were undergoing regular monitoring for their meningioma, with the reminder awaiting treatment.

Of the 116 Panel 1 registrants, all completed Round 1 and 96% completed Round 2 (4% attrition rate between rounds). Of the 32 Panel 2 registrants, 81% completed Round 1 but only 56% completed Round 2 (31% attrition rate between rounds). A summary of this data is provided in Table 2.

**Table 2.** COSMIC: Observation Registration and Survey Completion Data Combined with Panels 1 and 2 Participant Characteristic Data

Panel 1—Healthcare professionals & researchers	No.	Panel 2—Patients and those in caring/supporting roles	No.
			
**Registration & completion**		**Registration & completion**	
Registered	116	Registered	32
Completed Round 1	116 (100.0)	Completed Round 1	26 (81.3)
Completed Round 2	111 (95.7)	Completed Round 2	18 (56.3)
R1 to R2 attrition No. (% of those completing Round 1)	5 (4.3)	R1 to R2 attrition No. (% of those completing Round 1)	8 (30.8)
			
**Role/s within this stakeholder group**		**Role/s within this stakeholder group**	
Member of clinical team directly responsible for meningioma patients	61 (52.6)	Patient with a meningioma who has not undergone treatment	29 (90.6)
Researcher involved in meningioma research	9 (7.8)	Carer/family member to meningioma patient (untreated)	1 (3.2)
Both a member of a clinical team and a researcher as defined above	46 (39.7)	Charity/support group representative to meningioma patients (untreated)	2 (6.3)
			
**Years in meningioma relevant practice/research**		**No. years with diagnosis or time spent in caring/supporting role**	
		Less than 2	11 (34.4)
Less than 5	26 (22.4)	2 to 4	12 (37.5)
5 to 9	26 (22.4)	5 to 9	6 (18.8)
10 to 14	29 (25.0)	10 to 14	2 (6.3)
15 to 20	22 (19.0)	15 to 19	1 (3.2)
20 or more	13 (11.2)	20 or more	0 (0.0)
			
**Gender**		**Gender**	
Male	90 (77.6)	Female	28 (87.5)
Female	24 (20.7)	Male	4 (12.5)
Prefer not to say	2 (1.7)	Prefer not to say	0 (0.0)
			
**Age**		**Age**	
18-24	6 (5.2)	18-24	0 (0.0)
25-29	9 (7.8)	25-29	1 (3.2)
30-34	11 (9.5)	30-34	1 (3.2)
35-39	18 (15.5)	35-39	1 (3.2)
40-44	22 (19.0)	40-44	1 (3.2)
45-49	24 (20.7)	45-49	3 (9.4)
50-54	15 (13.0)	50-54	7 (21.9)
55-59	7 (6.0)	55-59	3 (9.4)
60-64	2 (1.7)	60-64	7 (21.9)
65-69	1 (0.9)	65-69	0 (0.0)
70-74	1 (0.9)	70-74	6 (18.8)
75-79	0 (0.0)	75-79	2 (6.3)
			
**Country of residence**		**Country of residence**	
United Kingdom	63 (54.3)	United Kingdom	20 (62.5)
United States	14 (12.1)	United States	9 (28.1)
Germany	10 (8.6)	Australia	2 (6.3)
Canada	4 (3.4)	Canada	1 (3.2)
Switzerland	3 (2.6)		
India	5 (4.3)		
Netherlands	2 (1.7)		
Italy	2 (1.7)		
Other (frequency less than 2)	13 (11.2)		
			
**Primary job role**		**Monitoring (scan) and awaiting treatment status (% of *n* = 29)**	
Neurosurgeon	80 (69.0)	I undergo regular monitoring (scan)	25 (86.2)
Oncologist (Clinical/Medical/Radiation)	10 (8.6)	I undergo regular monitoring (scan) and I am awaiting treatment	4 (13.9)
Researcher	8 (6.9)		
Neurologist	8 (6.9)		
Neuroradiologist	4 (3.4)		
Neuro-oncology/Skull base specialist nurse	3 (2.6)		
Neuropathologist	1 (0.9)		
Other	2 (1.7)		

### COSMIC Observation eDelphi Results Summary

After Round 1, the percentage of voting responses that were 7-9 for each outcome was calculated. Within Panel 1, 8 of the 17 outcomes presented had at least 80% of voting responses rated 7-9. Within Panel 2, this was 13 of 17 outcomes presented. A summary of this data is provided in Supplementary Appendix 6.

Twenty-two additional outcomes were proposed for addition to Round 2 by participants. These were reviewed, presented, and discussed with the same SMG members. No outcomes were added to Round 2. A summary of outcomes proposed for addition and reasons for not adding is provided in Supplementary Appendix 8.

Following Round 2, the percentage of voting responses that were 7-9 for each outcome was again calculated. Within Panel 1, 11 of the 17 outcomes presented had at least 80% of voting responses rated 7-9. Within Panel 2, this was 16 of 17 outcomes presented. Eleven outcomes met the definition for inclusion in the COSMIC: Observation COS at this stage. No outcomes met the definition for exclusion at this stage. Six outcomes remained and were progressed to the COSMIC: Observation consensus meeting. A summary of this data is provided in Supplementary Appendix 6.

## Phase 2b—Finalization of Core Outcome Sets with Consensus Meetings

### COSMIC: Intervention Consensus Meeting

#### Invited participants, attendees, and characteristics

Thirty participants agreed to attend the COSMIC: Intervention online consensus meeting that was held on Monday 10 October 2022.

From Panel 1, 6 participants from an expected 15 attended the meeting. This panel consisted of 3 neurosurgeons, a neurologist, an oncologist, and a neuroscience physiotherapist. From Panel 2, 11 participants from an expected 15 attended the meeting. This panel consisted of 9 patients, a family member of a patient, and a charity group representative. A summary of participants who agreed to attend this consensus meeting is provided in Supplementary Appendix 9.

#### COSMIC: Intervention consensus meeting results

Ten items were discussed at the consensus meeting. The first question concerned a proposal to merge 2 outcomes included in the COSMIC: Intervention COS following conclusion of the eDelphi survey as it was felt that the former “disease progression,” was an element of the latter “response to treatment.” A binary response was requested from all participants, and 100% from Panels 1 and 2 voted to merge the 2 included outcomes, resulting in a single outcome, “response to treatment” being included in the final COSMIC: Intervention COS. Items 2 through 9 required discussion and voting on the importance of the inclusion of outcomes not achieving a consensus decision following conclusion of the COSMIC: Intervention eDelphi survey. Two of these (questions 5 and 7) required a second round of discussion and voting. Question 7 remained without a consensus decision after the second round of discussion and voting (How important is it that the outcome “seizure control” is included in the COS?), and so a summary of the discussion points was provided via email following the conclusion of the meeting. Email votes were returned, and a consensus decision was achieved. The final question asked if the proposed final COSMIC: Intervention COS should be accepted, and 100% of participants from Panels 1 and 2 rated (7-9), thereby supporting its acceptance. A summary of the COSMIC: Intervention consensus meeting questions and vote results are summarized in Supplementary Appendix 11.

### COSMIC: Observation Consensus Meeting

#### Invited participants, attendees, and characteristics—.

Seventeen participants agreed to attend the COSMIC: Observation online consensus meeting that was held on Tuesday 11 October 2022.

From Panel 1, 9 participants from an expected 11 attended the meeting. This panel consisted of 3 neurosurgeons, a neurologist, 2 oncologists, 2 neuroradiologists, and a specialist nurse. From Panel 2, all 6 expected participants attended the meeting. This panel consisted of 5 patients and a charity group representative. A summary of participants who agreed to attend this consensus meeting is provided in Supplementary Appendix 10.

#### COSMIC: Observation consensus meeting results—.

Seven questions were posed to consensus meeting participants for discussion and voting. Questions 1 through 6 required discussion and voting on the importance of the inclusion of outcomes not achieving a consensus decision following conclusion of the COSMIC: Observation eDelphi survey. Five of these (questions 1, 3, 4, 5, and 6) required a second round of discussion and voting. Question 1 remained without a consensus decision after the second round of discussion and voting, and the meeting chair proposed that the SMG should resolve this following the conclusion of the meeting. The outcome in question was “tumor size” which had been dropped from the COSMIC: Intervention COS due to redundancy, and so the SMG decided to drop this outcome from the COSMIC: Observation COS also. The final question asked if the proposed final COSMIC: Observation COS should be accepted, and 100% of participants from Panels 1 and 2 rated (7-9), thereby supporting its acceptance. A summary of the COSMIC: Observation consensus meeting questions and vote results are summarized in Supplementary Appendix 12. The final COSMIC: Observation COS is presented in Table 3.

**Table 3.** The Final COSMIC: Intervention and COSMIC: Observation COS Grouped by Outcome Areas

Outcome area	COSMIC: Intervention COS	COSMIC: Observation COS
Mortality and survival	Surgical mortality Progression-free survival Meningioma-specific survival Overall survival	Growth-free survival Progression-free survival Meningioma-specific survival Overall survival
Progression and response	Tumor growth Response to treatment	Tumor growth Neurological signs Neurological symptoms Disease progression
Functioning and well-being	Physical functioning Emotional functioning Neurocognitive functioning Overall quality of life	Physical functioning Social functioning Role functioning Emotional functioning Neurocognitive functioning Overall quality of life
Resource use	Need for further treatment	Continue under active-surveillance
Treatment and adverse events	Extent of meningioma resection Adverse events of anti-tumor Treatment neurologic symptom Burden after treatment Neurologic status after treatment	Treatment given

## Discussion

The COSMIC Project has developed 2 COS for use in future meningioma clinical trials and studies through the application of rigorous methodological processes, involving a broad variety of international stakeholders. COSMIC: Intervention includes 15 outcomes and COSMIC: Observation includes 16 outcomes. Both contain outcomes across a range of outcome areas, including mortality, survival, progression, response, functioning and well-being, resource use, treatment, and adverse events. Eight core outcomes were common to both COS (tumor growth, physical, emotional, and neurocognitive functioning, overall quality of life, progression-free survival, meningioma-specific mortality and overall survival). Role and social functioning were core outcomes in COSMIC: Observation but not COSMIC: Intervention. Uptake in future meningioma clinical trials and studies can ensure that stakeholder-determined, critically important outcomes are consistently measured and reported.

### COS Development Process Highlights

We formed an international SAG including disease and methodological experts, but also PRPs who were instrumental in providing patient perspectives on the collaborative decisions that were made. Taken together, we are confident that the decision-making that has taken place has been representative and unbiased.

Considerable thought was given to scope, which resulted in developing 2 COS. In doing so, we have respected fundamental differences between those patients who have received treatment and patients who have not. These 2 populations, broadly speaking, would not be eligible for the same types of clinical studies.

We performed 2 thorough, but pragmatic systematic literature reviews which provided us with data from which we could develop 2 rationalized eDelphi survey long-lists. These actions were performed simultaneously to ensure consistency between both. We also launched both eDelphi surveys simultaneously and commenced a global recruitment drive via key national and international academic and charitable organizations, along with social media forums that engaged patients and their families. Patients were directed to register for 1 survey only (dependent on treatment history), whilst most other stakeholders were encouraged to register and complete both surveys. The high participation rate is evidence of a successful recruitment campaign.

Finally, we successfully conducted 2 online, international consensus meetings that were largely well attended, successful in their objectives, but also financially justifiable to funders and time-efficient for participants.

### Limitations—eDelphi Surveys

Whilst we are confident that our systematic reviews are methodologically sound and comprehensive, there still exists multiple layers of subjective data extraction, analysis, and rationalization required to generate the final eDelphi survey long-lists from primary literature sources.

We chose not to perform semi-structured interviews with meningioma patients, which may have identified additional outcomes not evident in the literature. However, we did provide eDelphi participants with the opportunity to suggest outcomes to add to Round 2 of the surveys, but most of these were not added.

The COSMIC: Observation eDelphi survey certainly had fewer participants, particularly from Panel 2 (which also had a high Round 1 to Round 2 attrition rate). Panel 1 participants from COSMIC: Intervention were encouraged to register and complete COSMIC: Observation also, and there existed multiple methods to promote this during the study. Inevitably, not all eligible Panel 1 participants registered to complete COSMIC: Observation. The low number of Panel 2 participants recruited to the COSMIC: Observation eDelphi survey may reflect our overall recruitment strategy. We recruited openly through several forums, including charitable organizations, mailing lists, and social media platforms, rather than direct contact with patients identified through institutional medical records. It is possible that active involvement with such forums is less for the incidental/untreated meningioma community. We recruited a few family and support workers into both surveys, despite making it clear in all communications that we wished for people to encourage additional survey completions.

### Limitations—Consensus Meetings

There were too many COSMIC: Intervention Panel 1 participants who did not attend the online consensus meeting as planned. This was not seen amongst Panel 2 participants. Despite the best intentions of Panel 1 participants, it is likely that the lack of a requirement to be physically present allowed other tasks and responsibilities to be prioritized. This is undoubtedly a limitation of conducting online, as opposed to in-person consensus meetings.

Reflecting the lack of eDelphi participants identifying as family and support group workers in both surveys, this of course translated across into those eligible for invitation to the consensus meetings from these stakeholder groups. We successfully obtained representation in both meetings but did need to invite some participants to both.

The authors acknowledge that there has been a delay from the date of the final consensus meetings to the publication of the final manuscript.

### How to Use these COS and Future Work

Both COS define *what* outcomes should be measured and reported in future meningioma clinical studies. More specifically, the COSMIC: Intervention COS is for use in clinical trials evaluating the effectiveness of treatment options for patients with an intracranial meningioma. However, COSMIC: Observation is for use in clinical studies that evaluate monitoring strategies for patients who have not been treated and may or may not need treatment in the future. Role and social functioning were included in this COS but not COSMIC: Intervention, and this may reflect higher levels of anxiety and uncertainty in those with an incidental meningioma which warrants assessment. There are scenarios where both COS could be used together if necessary. For example, a clinical trial that evaluates a monitoring strategy, followed by one or more interventions if predetermined criteria are met.

At this stage, *how* each outcome is to be measured is yet to be determined. This will require further consensus work to select definitions for binary and time to event outcomes (eg, overall survival), measurement methods or instruments for other outcomes (eg, response to treatment), or combinations of both (eg, progression-free survival). Finally, it is important to state that neither COS is required to exist statically. As treatment modalities change, so do priorities, and so this work should be revised and evolve with advancements that arise in the care of patients with intracranial meningioma.

## Supplementary material

Supplementary material is available online at *Neuro-Oncology Practice* (https://academic.oup.com/nop/).

npaf023_suppl_Supplementary_Appendixs

## FOOTNOTES

**International Consortium on Meningioma**: Kenneth Aldape, Karolyn Au, Jill Barnhartz-Sloan, Wenya Linda Bi, Felix Behling, Eelke M. Bos, Priscilla K. Brastianos, Chaya Brodie, Nicholas Butowski, Carlos Carlotti, Ana Castro, Aaron Cohen-Gadol, Marta Couce, Michael D. Cusimano, Maximilian Y. Deng, Francesco DiMeco, Katharine Drummond, Ian F. Dunn, Felix Ehret, Craig Erker, Michelle Felicella, Daniel M. Fountain, Evanthia Galanis, Norbert Galldiks, Caterina Giannini, Roland Goldbrunner, Brent Griffith, Rintaro Hashizume, C. Oliver Hanemann, Christel Herold-Mende, Luke Hnenny, Craig Horbinski, Raymond Y. Huang, Abdurrahman I. Islim, David James, Michael D. Jenkinson, Christine Jungk, Gerhard Jungwirth, Timothy J. Kaufmann, Boris Krischek, Sylvia Kurz, Daniel Lachance, Christian Lafougère, Katrin Lamszus, Alexander P. Landry, Ian Lee, Jeff C. Liu, Sybren L.N. Maas, Serge Makarenko, Tathiana Malta, Yasin Mamatjan, Alireza Mansouri, Christian Mawrin, Michael McDermott, Christopher P. Millward, Jennifer Moliterno-Gunel, Andrew Morokoff, David Munoz, Farshad Nassiri, Houtan Noushmehr, Ho-Keung Ng, Arie Perry, Farhad Pirouzmand, Laila M Poisson, Bianca Pollo, Aditya Ragunathan, David R. Raleigh, Mirjam Renovanz, Franz Ricklefs, Felix Sahm, Andrea Saladino, Antonio Santacroce, Thomas Santarius, Jens Schittenhelm, Christian Schichor, David Schultz, Nils O. Schmidt, Warren Selman, Helen Shih, Andrew Sloan, Julian Spears, Matija Snuderl, James Snyder, Suganth Suppiah, Erik Sulman, Ghazaleh Tabatabai, Marcos Tatagiba, Marco Timmer, Daniela Tirapelli, Joerg C. Tonn, Derek Tsang, Michael A. Vogelbaum, Andreas von Deimling, Tobias Walbert, Simon Walling, Justin Z. Wang, Patrick Y. Wen, Manfred Westphal, Adriana M. Workewych, Stephen Yip, Gabriel Zada, Gelareh Zadeh, Viktor Zherebitskiy.

**COSMIC: Intervention Collaborative:** Aaron A. Cohen-Gadol, Aaron Lawson McLean, Abdallah Groshi, Abdurrahman I. Islim, Abigail L. Clynch, Adel Helmy, Adrian Van Klaveren, Ahmad M. S. Ali, Ahmad Ozair, Ahmed Abougamil, Ahmed Sayed Kamal Aly, Aileen Watt, Alfonso Cerase, Ali Bakhsh, Amanda Stevens, Amir H. Zamanipoor Najafabadi, Andrea Saladino, Andrew Alalade, Andy Tudor, Angelos Kolias, Ann Barker, Anne Hutton, Arie Perry, Arjumand Faruqi, Asgeir S. Jakola, Ashwin Kumaria, Atulya A. Khosla, Benjamin Fisher, Beth Hawley, Betty Dalessandro, Carol Harrison, Carolina Benjamin, Caroline Crofts, Caroline Hayhurst, Catherine Strauss, Christine Jungk, Colin Watts, Corrine Dennis, Damien C. Weber, Daniel C. Walsh, David Bennett, David D.A. Lawson, David Mathieu, Debbie Barrett, Denise Wetzler, Desley Wilson, Dimitri Vanhauwaert, Dimitrios Paraskevopoulos, Donald Macarthur, Douglas Guedes de Castro, Edel Meagher, Edward McKintosh, Elisabeth Wightman, Elizabeth Baur, Erica Beaumont, Erik P. Sulman, Fardad T. Afshari, Felix Behling, Fenella Van Vliet, Fiona Anderson, Florencia Yorio, Florian Ringel, George E. Richardson, Gerhard Jungwirth, Gillian Taggart, Gonçalo Januário, Hani Marcus, Heather Mee, Helen Bulbeck, Hellene Boon, Herwin Speckter, Hugo Layard Horsfall, Husam Georges, Ian F. Dunn, Ian Lee, Irene Lawson, Ivo W. Tremont-Lukats, Jacob C.M. Low, James Galea, James M. Snyder, Jason P. Sheehan, Jerome J. Graber, Jillian Davis, Jillian Sokratous, Jo-Ann Howell, John C. Duddy, John Goodden, Jonathan Poots, Joy Roach, Julian Cahill, Karolyn Au, Katharine Drummond, Kathrin Whitehouse, Katrin Lamszus, Konstantina Karabatsou, Lara Thompson, Laura Darby, Laura Gerber, Lauren Harris, Lee Dawson, Leland Rogers, Lisa Bird, Liz Love, Louise Callaway, Magda Robertson, Mai Bishr, Marco Timmer, Martin Cunningham, Matthew G. Stovell, Menaka Paranathala, Michael Hardman, Michael Kosmin, Michael W. McDermott, Michael Weller, Michele Pappas, Mohammad A. Mustafa, Mohsen Javadpour, Nicholas Butowski, Nico Teske, Nils O. Schmidt, Nisaharan Srikandarajah, Norbert Galldiks, Patrick Nicholson, Patsy Keeler, Paul Boutros, Paul M. Brennan, Paul Grundy, Philipp Karschnia, Phillip Copley, Polly W. Blake, Ramesh Chelvarajah, Ramez W. Kirollos, Rasheed Zakaria, Raymond Huang, Rebecca Jackson, Roland Goldbrunner, Ronald J. Benveniste, Rosie Toal, Rowan E. Moore, Ruichong Ma, Ruman Rahman, Ryan K. Mathew, Ryojo Akagami, Sabrina Bell, Sandra Badham, Sanjeeva Jeyaretna, Sara Cooper, Sara McManus, Saurabh Sinha, Scott Rutherford, Sharon M. Mottola, Sharon Springall, Shaveta Mehta, Simon Harrison, Simon Lammy, Sogha Khawari, Sophie L. Zaretto, Stefan Nowicki, Stephen J. Price, Stephen T. Magill, Stephen Yip, Steve Braunstein, Stuart Smith, Stylianos Pikis, Susa Addis, Susan Chatburn, Tammy Norton, Terrie Cooper, Thomas Santarius, Tiffany Manzella, Tiit Mathiesen, Tobias Walbert, Torstein R. Meling, Tracy Andrew-Williams, Veronika Szasz-Kovacs, Victoria Turnbull, Vincent Lubrano, Vivek Bhat.

**COSMIC: Observation Collaborative:** Aaron A. Cohen-Gadol, Aaron Lawson McLean, Abdurrahman I. Islim, Abigail L. Clynch, Adel Helmy, Ahmad M. S. Ali, Ahmad Ozair, Ahmed Abougamil, Ahmed Sayed Kamal Aly, Alan John Dabbs, Alejo Stark, Alfonso Cerase, Amir H. Zamanipoor Najafabadi, Andrea Saladino, Andrew Alalade, Angelos Kolias, Arjumand Faruqi, Asgeir S. Jakola, Ashwin Kumaria, Atulya A.lawson Khosla, Bente S. Skeie, C Oliver Hanemann, Carolina Benjamin, Caroline Hayhurst, Charles Davis, Christine Jungk, Christine Olsen, Colin Watts, Damian Holliman, David Bennett, David D.A. Lawson, David Mathieu, Dimitri Vanhauwaert, Dimitrios Paraskevopoulos, Douglas Guedes de Castro, Edward McKintosh, Emilie Le Rhun, Erica Beaumont, Eva Quintero, Fardad T. Afshari, Felix Behling, Florencia Yorio, Gabrielle Goodchild, George E. Richardson, Gerhard Jungwirth, Gillie Marshall-Howells, Hani Marcus, Helen Bulbeck, Hugo Layard Horsfall, Husam Georges, Ian F. Dunn, Ian Lee, Ivo W. Tremont-Lukats, Jacob C.M. Low, Jane Dabbs, Jason P. Sheehan, Jerome J. Graber, Jillian Davis, Jillian Sokratous, Joelle Evans, John Goodden, Jonathan Poots, Joy Roach, Julian Cahill, Julie Freeman, Karolyn Au, Katharine Drummond, Kathrin Whitehouse, Lauren Harris, Mai Bishr, Marco Timmer, Marcos Tatagiba, Margaret Merry, Matthew G. Stovell, Menaka Paranathala, Michael Kosmin, Michael Weller, Mohsen Javadpour, Nico Teske, Nils O. Schmidt, Nisaharan Srikandarajah, Norbert Galldiks, Patrick Nicholson, Paul Boutros, Paul M. Brennan, Paul Grundy, Philipp Karschnia, Phillip Copley, Ramesh Chelvarajah, Ramez W. Kirollos, Rasheed Zakaria, Raymond Huang, Ruichong Ma, Ruman Rahman, Ryan K. Mathew, Sabrina Bell, Sally-Ann Price, Sanjeeva Jeyaretna, Sara Cooper, Saurabh Sinha, Scott Rutherford, Shaveta Mehta, Shelli Koszdin, Sogha Khawari, Stephen J. Price, Stephen T. Magill, Steve Braunstein, Stuart Smith, Stylianos Pikis, Susan Balding, Thomas Santarius, Tiit Mathiesen, Tobias Walbert, Torstein R. Meling, Vincent Lubrano, Vivek Bhat.

## Data Availability

All data generated in this study are available in the supplementary files. The data is available for use upon reasonable request from the corresponding author.
