# Supplementary material for: Core Outcome Sets for Meningioma In Clinical studies (COSMIC): An international patient and healthcare professional consensus for research studies
Source: Neurooncol Pract. 2025 Feb 20;12(4):700–13. doi: 10.1093/nop/npaf023 (PMC12349768; doi:10.1093/nop/npaf023)
Supplement: npaf023_suppl_Supplementary_Appendixs [file npaf023_suppl_supplementary_appendixs.docx]

Supplementary appendix 1 – COSMIC: Intervention SR long-list to eDelphi survey long-list conversion summary.

| **COMET outcome area** | **COMET outcome domain & No.** | **Standardised outcome term from systematic review** | **Fate** | **New name for eDelphi survey** |
| --- | --- | --- | --- | --- |
| Death | Mortality/survival (1) | death from pharmacotherapy | RATIONALIZED | adverse events of anti-tumor treatment |
|  |  | further intervention-free survival | REMOVED |  |
|  |  | HRQoL deterioration-free survival | RATIONALIZED | overall quality of life |
|  |  | meningioma-specific mortality | RETAINED |  |
|  |  | overall survival | RETAINED |  |
|  |  | perioperative mortality | RETAINED | surgical mortality |
|  |  | progression-free survival | RETAINED |  |
|  |  | recurrence-free survival | RATIONALIZED | progression-free survival |
| Physiological/clinical | Blood & lymphatic system (2) | anemia after surgery | RATIONALIZED | adverse events of anti-tumor treatment |
|  |  | clotting function after surgery | RATIONALIZED | adverse events of anti-tumor treatment |
|  |  | haematological adverse events from pharmacotherapy | RATIONALIZED | adverse events of anti-tumor treatment |
|  |  | need for blood transfusion | RATIONALIZED | adverse events of anti-tumor treatment |
|  |  | need for clotting factors | RATIONALIZED | adverse events of anti-tumor treatment |
|  |  | need for platelet transfusion | RATIONALIZED | adverse events of anti-tumor treatment |
|  | Cardiac (3) | cardiac adverse events from pharmacotherapy | RATIONALIZED | adverse events of anti-tumor treatment |
|  | Endocrine (5) | endocrine adverse events from pharmacotherapy | RATIONALIZED | adverse events of anti-tumor treatment |
|  | Ear & labyrinth (6) | ear adverse events after radiotherapy | RATIONALIZED | adverse events of anti-tumor treatment |
|  |  | ear adverse events from pharmacotherapy | RATIONALIZED | adverse events of anti-tumor treatment |
|  | Eye (7) | eye adverse events after radiotherapy | RATIONALIZED | adverse events of anti-tumor treatment |
|  |  | eye adverse events from pharmacotherapy | RATIONALIZED | adverse events of anti-tumor treatment |
|  | Gastrointestinal (8) | gastrointestinal adverse events after radiotherapy | RATIONALIZED | adverse events of anti-tumor treatment |
|  |  | gastrointestinal adverse events from pharmacotherapy | RATIONALIZED | adverse events of anti-tumor treatment |
|  |  | postoperative nausea | RATIONALIZED | adverse events of anti-tumor treatment |
|  |  | postoperative vomiting | RATIONALIZED | adverse events of anti-tumor treatment |
|  | General (9) | fatigue from pharmacotherapy | RATIONALIZED | adverse events of anti-tumor treatment |
|  |  | injection site reaction from pharmacotherapy | RATIONALIZED | adverse events of anti-tumor treatment |
|  |  | pain after radiotherapy | RATIONALIZED | adverse events of anti-tumor treatment |
|  |  | pain from pharmacotherapy | RATIONALIZED | adverse events of anti-tumor treatment |
|  |  | weight loss from pharmacotherapy | RATIONALIZED | adverse events of anti-tumor treatment |
|  | Hepatobilliary (10) | hepatobillary adverse events from pharmacotherapy | RATIONALIZED | adverse events of anti-tumor treatment |
|  | Immune system (11) | allergic reaction from pharmacotherapy | RATIONALIZED | adverse events of anti-tumor treatment |
|  |  | immunological response to pharmacotherapy | RATIONALIZED | response to treatment |
|  | Infection & infestation (12) | infection after radiotherapy | RATIONALIZED | adverse events of anti-tumor treatment |
|  |  | infection from pharmacotherapy | RATIONALIZED | adverse events of anti-tumor treatment |
|  |  | postoperative wound infection | RATIONALIZED | adverse events of anti-tumor treatment |
|  | Metabolism & nutrition (14) | electrolyte status after surgery | RATIONALIZED | adverse events of anti-tumor treatment |
|  |  | metabolic and nutrition adverse events from pharmacotherapy | RATIONALIZED | adverse events of anti-tumor treatment |
|  | Musculoskeletal &  connective tissue (15) | musculoskeletal and connective tissue adverse events from pharmacotherapy | RATIONALIZED | adverse events of anti-tumor treatment |
|  | Nervous system (17) | absolute growth rate | RATIONALIZED | tumor growth |
|  |  | ataxia after radiotherapy | RATIONALIZED | adverse events of anti-tumor treatment |
|  |  | blood loss | RATIONALIZED | adverse events during surgery |
|  |  | central nervous system necrosis after radiotherapy | RATIONALIZED | adverse events of anti-tumor treatment |
|  |  | cerebrospinal fluid leakage after radiotherapy | RATIONALIZED | adverse events of anti-tumor treatment |
|  |  | cognitive disturbance after radiotherapy | RATIONALIZED | adverse events of anti-tumor treatment |
|  |  | complete response | RATIONALIZED | response to treatment |
|  |  | cranial nerve dysfunction after radiotherapy | RATIONALIZED | adverse events of anti-tumor treatment |
|  |  | diagnostic efficacy of 5-ALA during surgery | REMOVED |  |
|  |  | disordered speech after radiotherapy | RATIONALIZED | adverse events of anti-tumor treatment |
|  |  | distribution of embolization agent | REMOVED |  |
|  |  | duration of surgery | RETAINED |  |
|  |  | edema cerebral after radiotherapy | RATIONALIZED | adverse events of anti-tumor treatment |
|  |  | encephalopathy after radiotherapy | RATIONALIZED | adverse events of anti-tumor treatment |
|  |  | extent of resection | RETAINED | extent of meningioma resection |
|  |  | haemostasis of surgical field during surgery | RATIONALIZED | adverse events during surgery |
|  |  | headache after radiotherapy | RATIONALIZED | adverse events of anti-tumor treatment |
|  |  | maximum 2D size of the tumor | RATIONALIZED | tumor size |
|  |  | minor response | RATIONALIZED | response to treatment |
|  |  | misplaced radiotherapy seed | REMOVED |  |
|  |  | nervous system adverse events from pharmacotherapy | RATIONALIZED | adverse events of anti-tumor treatment |
|  |  | neurologic status after treatment | RETAINED |  |
|  |  | neurologic symptom burden after treatment | RETAINED |  |
|  |  | new postoperative neurological deficit | RATIONALIZED | adverse events of anti-tumor treatment |
|  |  | partial response | RATIONALIZED | response to treatment |
|  |  | peripheral motor neuropathy after radiotherapy | RATIONALIZED | adverse events of anti-tumor treatment |
|  |  | peripheral sensory neuropathy after radiotherapy | RATIONALIZED | adverse events of anti-tumor treatment |
|  |  | pharmacotherapy requirements during surgery | RATIONALIZED | adverse events during surgery |
|  |  | physiological response to surgery | RATIONALIZED | adverse events during surgery |
|  |  | postoperative brain edema | RATIONALIZED | adverse events of anti-tumor treatment |
|  |  | postoperative cranial nerve deficit | RATIONALIZED | adverse events of anti-tumor treatment |
|  |  | postoperative dysphasia | RATIONALIZED | adverse events of anti-tumor treatment |
|  |  | postoperative hydrocephalus | RATIONALIZED | adverse events of anti-tumor treatment |
|  |  | postoperative impotence | REMOVED |  |
|  |  | postoperative intracranial hemorrhage | RATIONALIZED | adverse events of anti-tumor treatment |
|  |  | postoperative limb weakness | RATIONALIZED | adverse events of anti-tumor treatment |
|  |  | postoperative pain | RATIONALIZED | adverse events of anti-tumor treatment |
|  |  | postoperative pseudomeningocele | RATIONALIZED | adverse events of anti-tumor treatment |
|  |  | postoperative seizure | RATIONALIZED | adverse events of anti-tumor treatment |
|  |  | postoperative somnolence | RATIONALIZED | adverse events of anti-tumor treatment |
|  |  | postoperative visual impairment | RATIONALIZED | adverse events of anti-tumor treatment |
|  |  | progressive disease | RATIONALIZED | response to treatment |
|  |  | radiographic response to treatment | RATIONALIZED | response to treatment |
|  |  | radiological response to embolization | RATIONALIZED | response to treatment |
|  |  | radiological response to interstitial radiotherapy | RATIONALIZED | response to treatment |
|  |  | relative growth rate | RATIONALIZED | tumor growth |
|  |  | seizure after radiotherapy | RATIONALIZED | adverse events of anti-tumor treatment |
|  |  | somnolence after radiotherapy | RATIONALIZED | adverse events of anti-tumor treatment |
|  |  | stable disease | RATIONALIZED | response to treatment |
|  |  | stroke after radiotherapy | RATIONALIZED | adverse events of anti-tumor treatment |
|  |  | syndrome of the trephined | RATIONALIZED | adverse events of anti-tumor treatment |
|  |  | tremor after radiotherapy | RATIONALIZED | adverse events of anti-tumor treatment |
|  |  | volume of tumor | RATIONALIZED | tumor size |
|  | Renal and urinary (19) | renal and urinary adverse events from pharmacotherapy | RATIONALIZED | adverse events of anti-tumor treatment |
|  | Reproductive system  & breast (20) | reproductive and breast adverse events from pharmacotherapy | RATIONALIZED | adverse events of anti-tumor treatment |
|  | Psychiatric (21) | psychiatric adverse events from pharmacotherapy | RATIONALIZED | adverse events of anti-tumor treatment |
|  |  | psychiatric disorder after radiotherapy | RATIONALIZED | adverse events of anti-tumor treatment |
|  | Respiratory, thoracic  & mediastinal (22) | respiratory adverse events from pharmacotherapy | RATIONALIZED | adverse events of anti-tumor treatment |
|  | Skin & subcutaneous tissue (23) | skin adverse events after radiotherapy | RATIONALIZED | adverse events of anti-tumor treatment |
|  |  | skin adverse events from pharmacotherapy | RATIONALIZED | adverse events of anti-tumor treatment |
|  | Vascular (24) | postoperative thromboembolic events | RATIONALIZED | adverse events of anti-tumor treatment |
|  |  | vascular adverse events from pharmacotherapy | RATIONALIZED | adverse events of anti-tumor treatment |
| Life impact | Functioning (all) (25-29) | health-related quality of life | RATIONALIZED | overall quality of life |
|  | Physical functioning (25) | physical functioning | RETAINED |  |
|  | Cognitive functioning (29) | neurocognitive functioning | RETAINED |  |
|  | Delivery of care (32) | discontinuation of pharmacotherapy due to adverse events | RATIONALIZED | withdrawal from trial |
|  |  | discontinuation of radiotherapy due to adverse events | RATIONALIZED | withdrawal from trial |
|  |  | reduction of pharmacotherapy dose due to adverse events | RATIONALIZED | adverse events of anti-tumor treatment |
|  |  | trial withdrawal - Clinician decision | RATIONALIZED | withdrawal from trial |
|  |  | trial withdrawal - Patient decision | RATIONALIZED | withdrawal from trial |
|  |  | unplanned return to theatre | RATIONALIZED | adverse events of anti-tumor treatment |
| Resource use | Hospital (35) | duration of hospital stay | RETAINED |  |
|  |  | duration of intensive care stay | RETAINED |  |
|  | Need for further intervention (36) | need for further intervention | RETAINED | need for further treatment |
| Adverse events | Adverse events/effects (38) | adverse events after radiotherapy | RATIONALIZED | adverse events of anti-tumor treatment |
|  |  | adverse events from pharmacotherapy | RATIONALIZED | adverse events of anti-tumor treatment |
|  |  | perioperative adverse events | RATIONALIZED | adverse events of anti-tumor treatment |

Supplementary appendix 2 – COSMIC: Observation SR long-list to eDelphi survey long-list conversion summary.

| **COMET outcome area** | **COMET outcome domain & No.** | **Standardised outcome term from systematic review** | **Fate** | **New name for eDelphi survey** |
| --- | --- | --- | --- | --- |
| Death | Mortality/survival (1) | growth-free survival | RETAINED |  |
|  |  | meningioma-specific mortality | RETAINED |  |
|  |  | non-meningioma-specific mortality | REMOVED |  |
|  |  | overall survival | RETAINED |  |
|  |  | progression-free survival | RETAINED |  |
| Physiological/clinical | Eye outcomes (7) | visual impairment | RATIONALIZED | neurological symptoms |
|  | General outcomes (9) | fatigue | RATIONALIZED | neurological symptoms |
|  | Nervous system outcomes (17) | absolute growth rate | RATIONALIZED | tumor growth |
|  |  | development or increase of oedema | RATIONALIZED | tumor growth |
|  |  | difficulty walking | RATIONALIZED | neurological symptoms |
|  |  | exponential growth pattern | RATIONALIZED | tumor growth |
|  |  | growth of tumor | RATIONALIZED | tumor growth |
|  |  | growth of tumor to greater than 10cm3 | RATIONALIZED | tumor growth |
|  |  | limb paralysis | RATIONALIZED | neurological symptoms |
|  |  | limb weakness | RATIONALIZED | neurological symptoms |
|  |  | linear growth pattern | RATIONALIZED | tumor growth |
|  |  | maximum 2D size of the tumor | RATIONALIZED | tumor size |
|  |  | maximum diameter of the tumor | RATIONALIZED | tumor size |
|  |  | need for any treatment | RATIONALIZED | treatment given |
|  |  | need for cerebrospinal fluid diversion | RATIONALIZED | treatment given |
|  |  | need for radiotherapy | RATIONALIZED | treatment given |
|  |  | need for stereotactic radiosurgery | RATIONALIZED | treatment given |
|  |  | need for surgery | RATIONALIZED | treatment given |
|  |  | need for surgery and radiotherapy | RATIONALIZED | treatment given |
|  |  | neurological signs | RETAINED |  |
|  |  | new symptoms | RATIONALIZED | neurological symptoms |
|  |  | no tumor growth | RATIONALIZED | tumor growth |
|  |  | parabolic growth pattern | RATIONALIZED | tumor growth |
|  |  | quasi-exponential growth pattern | RATIONALIZED | tumor growth |
|  |  | relative growth rate | RATIONALIZED | tumor growth |
|  |  | seizure | RATIONALIZED | neurological symptoms |
|  |  | sigmoid growth pattern | RATIONALIZED | tumor growth |
|  |  | symptom-free | RATIONALIZED | neurological symptoms |
|  |  | time to clinical progression | RATIONALIZED | disease progression |
|  |  | time to growth | RATIONALIZED | tumor growth |
|  |  | time to progression | RATIONALIZED | disease progression |
|  |  | tumor doubling time | RATIONALIZED | tumor growth |
|  |  | tumor invasion into venous sinus | RATIONALIZED | tumor growth |
|  |  | tumor regression | RATIONALIZED | tumor growth |
|  |  | volume of edema | RATIONALIZED | tumor size |
|  |  | volume of tumor | RATIONALIZED | tumor size |
|  |  | volume of tumor and edema | RATIONALIZED | tumor size |
|  |  | worsening symptoms | RATIONALIZED | neurological symptoms |
| Life impact | Physical functioning (25) | physical functioning | RETAINED |  |
|  | Emotional functioning/wellbeing (28) | emotional functioning | RETAINED |  |
|  | Cognitive functioning (29) | neurocognitive functioning | RETAINED |  |
|  | Global Quality of Life (30) | overall quality of life | RETAINED |  |
|  | Delivery of care (32) | continue under active-surveillance | RETAINED |  |
|  |  | discharged from active-surveillance | REMOVED |  |
|  |  | not able to undergo treatment | RATIONALIZED | treatment given |
|  |  | patient declined treatment | RATIONALIZED | treatment given |
|  |  | patient request for treatment | RETAINED |  |
|  |  | stereotactic radiosurgery no longer a treatment option | RATIONALIZED | treatment given |

Supplementary appendix 3 - COSMIC: Intervention eDelphi long-list, survey pages and lay definitions.

| **Delphi page** | **Outcome** | **Definition** |
| --- | --- | --- |
| Size & growth | tumor size | An estimate of the size of the meningioma on brain scans. We can use size to calculate growth and the rate of growth of the meningioma. |
|  | tumor growth | When we see a change in the size of the meningioma on brain scans. |
| Progression & Response | disease progression | A change in the patient’s health condition due to the meningioma (e.g. neurological signs, neurological symptoms, or a change in the meningioma on brain scans). |
|  | response to treatment | How the meningioma responds on brain scans during or after treatment (e.g. after surgery, radiotherapy, and/or drug therapy). |
| Treatment & Adverse Events | duration of surgery | The length of time required to complete meningioma surgery. |
|  | adverse events during surgery | An unwanted and unintended event during surgery for meningioma (e.g. excessive blood loss). |
|  | surgical mortality | Death occurring within 30 days after surgery in or out of the hospital. |
|  | extent of meningioma resection | An estimate of the amount of meningioma removed following surgery. |
|  | adverse events of anti-tumor treatment | An unwanted and unintended symptom, sign, disease or test result after treatment for meningioma (e.g. seizure, wound infection, fatigue…. After surgery, radiotherapy, and/or drug therapy). |
| Neurological status | neurologic symptom burden after treatment | The quantity and type of symptoms experienced by the patient after treatment for meningioma (e.g. fatigue, limb weakness, visual impairment). |
|  | neurologic status after treatment | An assessment of the overall status of the nervous system after treatment for meningioma (e.g. neurological signs and neurological symptoms). |
| Functioning & well-being | physical functioning | Impact of treatment for meningioma on physical activities of daily living (e.g. independence, self-care, performance status, motor skills, sexual dysfunction). |
|  | social functioning | Impact of treatment for meningioma on social functioning (e.g. ability to socialise, behaviour within society, communication, companionship, psychosocial development, aggression, participation). |
|  | role functioning | Impact of treatment for meningioma on role (e.g. ability to care for children, work status). |
|  | emotional functioning | Impact of treatment for meningioma on emotions or overall wellbeing (e.g. ability to cope, worry, frustration, confidence, psychological status, and stigma). |
|  | neurocognitive functioning | Impact of treatment for meningioma on neurocognitive function (e.g. memory problems, lack of concentration, attention deficit). |
|  | overall quality of life | Impact of meningioma on the overall quality of a patient’s life. |
| Mortality/survival | progression-free survival | The length of time that a patient lives with a meningioma after joining a trial, without it causing clinically relevant symptoms, signs, or changing on a brain scan. |
|  | meningioma-specific mortality | Deaths that occurred during the study period due to the meningioma. |
|  | overall survival | How long a patient with a meningioma lives for after joining a clinical trial. |
| Delivery of Care | withdrawal from trial | The number of patients dropping out of the clinical trial early (e.g. due to side-effects). |
| Resource use | duration of hospital stay | The length of time spent in hospital after treatment/intervention for meningioma. |
|  | duration of intensive care stay | The length of time spent in intensive care after treatment/intervention for meningioma. |
|  | need for further treatment | The need for additional treatment to control the meningioma, after completing the first stage of treatment (e.g. the need for further surgery after an initial trial which investigated surgery for meningioma). |

Supplementary appendix 4 - COSMIC: Observation eDelphi long-list, survey page and lay definitions.

| **Delphi page** | **Outcome** | **Definition** |
| --- | --- | --- |
| Size & growth | tumor size | An estimate of the size of the meningioma on brain scans. We can use size to calculate growth and the rate of growth of the meningioma. |
|  | tumor growth | When we see a change in the size of the meningioma on brain scans. |
| Progression | neurological signs | When a healthcare professional examines you during a clinical trial and finds abnormal neurological physical signs, (e.g. weakness of a limb, worsening vision, or impaired coordination). |
|  | neurological symptoms | When you experience symptoms that develop during a clinical trial, that may be related to the meningioma (e.g. weakness of a limb, worsening vision, or the occurrence of seizures). |
|  | disease progression | A change in the patient’s health condition due to the meningioma (e.g. neurological signs, neurological symptoms, or a change in the meningioma on brain scans). |
| Functioning & well-being | physical functioning | Impact of meningioma on physical activities of daily living (e.g. independence, self-care, performance status, motor skills, sexual dysfunction). |
|  | social functioning | Impact of meningioma on social functioning (e.g. ability to socialise, behaviour within society, communication, companionship, psychosocial development, aggression, participation). |
|  | role functioning | Impact of meningioma on role (e.g. ability to care for children, work status). |
|  | emotional functioning | Impact of meningioma on emotions or overall wellbeing (e.g. ability to cope, worry, frustration, confidence, psychological status, and stigma). |
|  | neurocognitive functioning | Impact of meningioma on neurocognitive function (e.g. memory problems, lack of concentration, attention deficit). |
|  | overall quality of life | Impact of meningioma on the overall quality of a patient’s life. |
| Mortality/survival | growth-free survival | The length of time that a patient lives with a meningioma after joining a trial, without growth being seen on a brain scan. |
|  | progression-free survival | The length of time that a patient lives with a meningioma after joining a trial, without it causing clinically relevant symptoms, signs, or changing on a brain scan. |
|  | meningioma-specific mortality | Deaths that occurred during the study period due to the meningioma. |
|  | overall survival | How long a patient with a meningioma lives for after joining a clinical trial. |
| Delivery of Care | treatment given | Treatment given for meningioma during the study period (e.g. surgery, stereotactic radiosurgery, radiotherapy, or other procedures including shunts to divert brain fluid). |
|  | continue under active-surveillance | The number of patients who remain under clinical follow-up by a clinical care team at the end of the study period. From this, the number of patients discharged from further follow-up can also be calculated. |

Supplementary appendix 5 - COSMIC: Intervention eDelphi voting results.

|  | **Percentage of voting responses rated 7-9 on Likert scale (No. responses)** | | | |  |
| --- | --- | --- | --- | --- | --- |
| **Outcome** | **eDelphi R1 P1 (n=147)** | **eDelphi R1 P2 (n=105)** | **eDelphi R2 P1 (n=147)** | **eDelphi R2 P2 (n=105)** | **eDelphi result** |
| tumor size | 65 (144) | 78 (91) | 67 (136) | 85 (68) | Undecided |
| tumor growth | 79 (144) | 92 (91) | 89 (136) | 93 (67) | Include |
| disease progression | 91 (145) | 93 (88) | 95 (135) | 96 (69) | Include |
| response to treatment | 91 (144) | 95 (88) | 93 (134) | 96 (69) | Include |
| duration of surgery | 9 (144) | 36 (88) | 9 (136) | 23 (69) | Exclude |
| adverse events during surgery | 57 (144) | 83 (88) | 62 (136) | 84 (69) | Undecided |
| surgical mortality | 92 (144) | 88 (88) | 93 (136) | 94 (69) | Include |
| extent of meningioma resection | 78 (144) | 82 (87) | 83 (136) | 84 (68) | Include |
| adverse events of anti-tumor treatment | 88 (144) | 84 (88) | 93 (136) | 87 (69) | Include |
| neurologic symptom burden after treatment | 88 (144) | 88 (89) | 91 (135) | 90 (67) | Include |
| neurologic status after treatment | 86 (144) | 91 (90) | 93 (135) | 93 (68) | Include |
| physical functioning | 85 (144) | 90 (88) | 88 (135) | 93 (71) | Include |
| social functioning | 65 (144) | 72 (88) | 67 (135) | 77 (69) | Undecided |
| role functioning | 58 (142) | 76 (87) | 65 (133) | 79 (68) | Undecided |
| emotional functioning | 60 (143) | 85 (88) | 66 (135) | 82 (68) | Undecided |
| neurocognitive functioning | 78 (144) | 94 (88) | 87 (135) | 94 (68) | Include |
| overall quality of life | 94 (144) | 94 (88) | 96 (135) | 97 (68) | Include |
| progression-free survival | 83 (144) | 84 (90) | 90 (136) | 88 (67) | Include |
| meningioma-specific mortality | 93 (144) | 91 (88) | 96 (135) | 96 (67) | Include |
| overall survival | 81 (144) | 93 (88) | 92 (135) | 97 (68) | Include |
| withdrawal from trial | 55 (141) | 51 (88) | 56 (135) | 52 (65) | Undecided |
| duration of hospital stay | 18 (146) | 20 (89) | 13 (134) | 15 (68) | Exclude |
| duration of intensive care stay | 23 (146) | 34 (89) | 16 (134) | 28 (68) | Exclude |
| need for further treatment | 72 (146) | 79 (89) | 76 (133) | 75 (68) | Undecided |
| seizure control | n/a | n/a | 70 (133) | 86 (66) | Undecided |

Supplementary appendix 6 - COSMIC: Observation eDelphi voting results.

|  | **Percentage of voting responses rated 7-9 on Likert scale (No. responses)** | | | |  |
| --- | --- | --- | --- | --- | --- |
| **Outcome** | **eDelphi R1 P1 (n=116)** | **eDelphi R1 P2 (n=32)** | **eDelphi R2 P1 (n=116)** | **eDelphi R2 P2 (n=32)** | **eDelphi result** |
| tumor size | 67 (116) | 79 (28) | 70 (111) | 83 (18) | Undecided |
| tumor growth | 87 (116) | 100 (27) | 93 (111) | 89 (18) | Include |
| neurological signs | 78 (116) | 88 (25) | 85 (111) | 88 (17) | Include |
| neurological symptoms | 85 (116) | 92 (26) | 94 (111) | 88 (17) | Include |
| disease progression | 91 (115) | 88 (25) | 95 (111) | 88 (17) | Include |
| physical functioning | 84 (116) | 86 (28) | 87 (111) | 94 (17) | Include |
| social functioning | 63 (116) | 79 (28) | 66 (111) | 88 (17) | Undecided |
| role functioning | 60 (115) | 79 (28) | 64 (111) | 100 (17) | Undecided |
| emotional functioning | 64 (115) | 86 (28) | 65 (111) | 88 (17) | Undecided |
| neurocognitive functioning | 72 (116) | 86 (28) | 78 (111) | 94 (17) | Undecided |
| overall quality of life | 89 (116) | 89 (28) | 95 (111) | 100 (17) | Include |
| growth-free survival | 61 (116) | 81 (26) | 69 (111) | 76 (17) | Undecided |
| progression-free survival | 79 (116) | 88 (26) | 88 (111) | 88 (17) | Include |
| meningioma-specific mortality | 91 (116) | 81 (26) | 95 (111) | 82 (17) | Include |
| overall survival | 80 (116) | 77 (26) | 93 (111) | 82 (17) | Include |
| treatment given | 83 (115) | 96 (24) | 89 (110) | 94 (16) | Include |
| continue under active-surveillance | 77 (116) | 88 (26) | 85 (111) | 94 (17) | Include |

Supplementary appendix 7 – COSMIC: Intervention eDelphi additional outcome decisions.

| **Outcomes proposed for addition after R1 by eDelphi participants** | **Include?** | **If yes, outcome added, if no. reason.** |
| --- | --- | --- |
| use of resources for SRS - time to plan; time to treat; complications like for surgery etc | no | Not an outcome applicable to study population |
| Support for rest of family | no | Not an outcome applicable to study population |
| I think the overall health; fitness and age of a meningioma patient should also be added. To see how much fitness; health; spirituality; etc add to treatment outcomes | no | Not an outcome - baseline characteristic |
| time to neurologic symptom decline (NANO?) | no | Synonymous with existing outcome - neurologic symptom burden |
| steroid use | no | Not an outcome – baseline characteristic |
| seizure control/freedom | yes | Addition - seizures |
| pain/headache | no | Synonymous with existing outcome - neurologic symptom burden after treatment |
| time to quality of life decline | no | Synonymous with existing outcome - overall quality of life |
| #ER visits/hospitalizations/subacute nursing/inpatient rehab facility days | no | Synonymous with existing outcome - adverse events of anti-tumor treatment |
| Risk of seizures | no | Not an outcome applicable to study population |
| Quality of care from Hospital overall | no | Not an outcome applicable to study population |
| availability and access to medical specialists and treatmenrs in relation to where you live | no | Not an outcome applicable to study population |
| Tumour grade | no | Not an outcome - baseline characteristic |
| Tumour location | no | Not an outcome - baseline characteristic |
| Meningioma grade | no | Not an outcome - baseline characteristic |
| Meningioma characteristics - brain invasion | no | Not an outcome - baseline characteristic |
| Meningioma characteristics - sinus invasion | no | Not an outcome - baseline characteristic |
| Meningioma characteristics - skull base/convexity/falcine/parafalcine/posterior fossa/ventricular | no | Not an outcome - baseline characteristic |
| Meningioma characteristics - cyst | no | Not an outcome - baseline characteristic |
| Meningioma characteristics - brain oedema | no | Not an outcome - baseline characteristic |
| Location Specific Outcome Metrics for skull base meningioma | no | Synonymous with existing outcome - neurologic status after treatment |
| Seizure control | yes | Addition - seizures |
| Follow up after 6 week post-op | no | Not an outcome - non-specific |
| Ophthalmological and endocrinological outcomes in those with anterior skull base meningioma | no | Synonymous with existing outcome - neurologic status after treatment |
| Fear of disease recurrence among patients | no | Synonymous with existing outcome - emotional functioning |
| Psychological distress among patients | no | Synonymous with existing outcome - emotional functioning |
| Neurological burden before surgery | no | Not an outcome - baseline characteristic |
| QoL before surgery | no | Not an outcome - baseline characteristic |
| meningioma texture (consistency; adherence; vascularization) | no | Not an outcome - baseline characteristic |
| requirement for subsequent treatment (rads/chemo/repeat surgery) | no | Synonymous with existing outcome - need for further treatment |
| Change of qol over time after treatment | no | Synonymous with existing outcome - overall quality of life |
| Access to neurological/physiotherapy support after surgery | no | Not an outcome applicable to study population |
| Symptoms to be acknowledged as a result of meningioma; regardless of what is learnt from text books/lecturers | no | Not an outcome - non-specific |
| Appearance of CSF leakage as particular complication after surgery of meningiomas | no | Synonymous with existing outcome - adverse events of anti-tumor treatment |
| Location/site of meningioma | no | Not an outcome - baseline characteristic |
| LONG term quality of life outcomes; as assessed by the patient. e.g. at 5;10; 15 and 20 years post treatment | no | Synonymous with existing outcome - overall quality of life |
| How well connected to medical services the patient feels - we're routinely just left for a year with no contact; which isn't good quality treatment of a lifelong condition | no | Not an outcome applicable to study population |
| Whether patient felt they were able to ask all their questions; both before and after treatment - e.g. if issues arose 2 years after treatment; was there someone to contact? | no | Not an outcome applicable to study population |
| Whether medical services took account of patient's personal circumstances (partner; income; kids; etc) | no | Not an outcome applicable to study population |
| More for the service rather than the patient: How does treatment follow up compare to best practice in other lifelong conditions? | no | Not an outcome - non-specific |
| Vitality | no | Synonymous with existing outcome - overall quality of life |
| Receptors in tumour ie oestrogen / progesterone | no | Not an outcome - baseline characteristic |
| Grading picture. Post op. Accuracy within tissue as some grade 1’s behave differently as mixed cell picture affects post op management | no | Not an outcome - baseline characteristic |
| Return to work | no | Synonymous with existing outcome - role functioning |
| Pre-operative neurological functioning | no | Not an outcome - baseline characteristic |
| Pre-operative wellbeing/QoL mesurement | no | Not an outcome - baseline characteristic |
| Frailty index | no | Synonymous with existing outcome - physical functioning |
| Physical disability after treatment including but not limited partial paralysis; neuropathy | no | Synonymous with existing outcome - neurologic status after treatment |
| Surgical team acknowledgement; commitment and support for post surgical side effects | no | Not an outcome - non-specific |
| Treatment modality specific metrics of experience/expertise of local team | no | Not an outcome - non-specific |
| Tumour grade | no | Not an outcome - baseline characteristic |
| Brain Invasion | no | Not an outcome - baseline characteristic |
| Symptom specifics as classified into headache; weakness; sensory; bladder/bowel; sexual dysfunction etc | no | Synonymous with existing outcome - neurologic symptom burden after treatment |
| Tumor location | no | Not an outcome - baseline characteristic |
| Genomic data | no | Not an outcome - baseline characteristic |
| Epigenetic data | no | Not an outcome - baseline characteristic |
| Metastasis free survival (for high-grade meningioma) | no | Synonymous with existing outcome - progression-free survival |
| WHO grading and malignant transformation | no | Not an outcome - baseline characteristic |
| Long term follow-up > 5 years | no | Not an outcome - non-specific |
| support and education for meningioma patients about what to expect so that symptoms are less frightening | no | Not an outcome applicable to study population |
| Tumor location (Convexity/Falx vs Skull Base vs Other) | no | Not an outcome - baseline characteristic |
| Patient experience of intervention | no | Not an outcome applicable to study population |
| Carer experience of intervention | no | Not an outcome applicable to study population |
| return to work | no | Synonymous with existing outcome - role functioning |
| causes of meningiomas | no | Not an outcome - non-specific |
| PROs | no | Not an outcome - non-specific |

Supplementary appendix 8 – COSMIC: Observation eDelphi additional outcome decisions.

| **Outcomes proposed for addition after R1 by eDelphi participants** | **Include?** | **If yes, outcome added, if no. reason.** |
| --- | --- | --- |
| Location of Tumour | no | Not an outcome - baseline characteristic |
| Deterioration free survival | no | Synonymous with existing outcome - progression-free survival |
| Intervention required | no | Synonymous with existing outcome - treatment given |
| Number of meningiomas | no | Not an outcome - baseline characteristic |
| Annual rate of growth (volume) | no | Specific metric of an existing outcome - tumor growth |
| Length of time of follow up | no | Not an outcome - Study descriptive |
| Change of treatment modality originally chosen (eg surveillance to SRS/surgery) | no | Synonymous with existing outcome - treatment given |
| Number of patients lost to follow up | no | Not an outcome - Study descriptive |
| Psychological distress related to tumour and observation/treatment | no | Synonymous with existing outcome - emotional functioning |
| Financial distress related to tumour and observation/treatment | no | Not an outcome applicable to study population |
| MR imaging characteristics | no | Not an outcome - baseline characteristic |
| location/site of meningioma | no | Not an outcome - baseline characteristic |
| Psychological care of patients following diagnosis | no | Synonymous with existing outcome - emotional functioning |
| Peritumoral focal edema (as parameter for fast tumor growth) | no | Synonymous with existing outcome - tumor growth |
| WHO grading if treatment is performed | no | Not an outcome - baseline characteristic |
| Presence of peritumoral brain edema | no | Synonymous with existing outcome - tumor growth |
| Tumor Location (Convexity/Falx; Skull Base; Other) | no | Not an outcome - baseline characteristic |
| Outcome | no | Not an outcome - non-specific |
| Reason(s) to opt for observation | no | Not an outcome - baseline characteristic |
| all cause (or non-meningioma related) mortality | no | Inverse of existing outcome - meningioma-specific mortality |
| Pathological Grade and Time to recurrence | no | Not an outcome - baseline characteristic, Not an outcome applicable to study population |
| Methylation Status and Time to recurrence | no | Not an outcome - baseline characteristic, Not an outcome applicable to study population |

Supplementary appendix 9 – COSMIC: Intervention attendees.

| **Participant No.** | **Gender** | **Role** | **Country** | **Status** |
| --- | --- | --- | --- | --- |
| 1 | Male | Oncologist (Clinical/Medical/Radiation) | Switzerland | DNA |
| 2 | Male | Oncologist (Clinical/Medical/Radiation) | United States of America | DNA |
| 3 | Female | Oncologist (Clinical/Medical/Radiation) | United Kingdom | Attended |
| 4 | Male | Neurosurgeon | United States of America | Attended |
| 5 | Female | Neurosurgeon | Australia | DNA |
| 6 | Female | Neurosurgeon | Germany | Attended |
| 7 | Male | Neurosurgeon | Denmark | DNA |
| 8 | Male | Neurosurgeon | United Kingdom | Attended |
| 9 | Female | Neuro-oncology/Skull base specialist nurse | United Kingdom | DNA |
| 10 | Female | Neurosciences Physiotherapist | United Kingdom | Attended |
| 11 | Male | Neurologist | United Kingdom | Attended |
| 12 | Male | Neurologist | United States of America | DNA |
| 13 | Male | Neuroradiologist | United States of America | DNA |
| 14 | Female | Neuroradiologist | United Kingdom | DNA |
| 15 | Male | Neuropathologist | Canada | DNA |
| 16 | Female | Patient | United States of America | Attended |
| 17 | Female | Patient | United Kingdom | Attended |
| 18 | Male | Patient | United Kingdom | Attended |
| 19 | Female | Patient | United States of America | DNA |
| 20 | Female | Patient | Australia | Attended |
| 21 | Female | Patient | United Kingdom | DNA |
| 22 | Male | Patient | United Kingdom | Attended |
| 23 | Female | Patient | United Kingdom | DNA |
| 24 | Female | Patient | United States of America | DNA |
| 25 | Female | Patient | France | Attended |
| 26 | Female | Patient | United Kingdom | Attended |
| 27 | Female | Patient | United Kingdom | Attended |
| 28 | Female | Patient | United States of America | Attended |
| 29 | Female | Carer/Family member | United Kingdom | Attended |
| 30 | Female | Charity/Support Group Representative | United Kingdom | Attended |

Supplementary appendix 10 – COSMIC: Observation attendees.

| **Participant No.** | **Gender** | **Role** | **Country** | **Status** |
| --- | --- | --- | --- | --- |
| 1 | Male | Oncologist (Clinical/Medical/Radiation) | United States of America | Attended |
| 2 | Female | Oncologist (Clinical/Medical/Radiation) | United Kingdom | Attended |
| 3 | Male | Neurosurgeon | United States of America | Attended |
| 4 | Female | Neurosurgeon | Australia | DNA |
| 5 | Female | Neurosurgeon | Norway | Attended |
| 6 | Male | Neurosurgeon | Denmark | Attended |
| 7 | Female | Neuro-oncology/Skull base specialist nurse | United Kingdom | Attended |
| 8 | Female | Neurosciences Physiotherapist | United Kingdom | DNA |
| 9 | Male | Neurologist | United States of America | Attended |
| 10 | Male | Neuroradiologist | United States of America | Attended |
| 11 | Female | Neuroradiologist | United Kingdom | Attended |
| 12 | Female | Patient | United Kingdom | Attended |
| 13 | Female | Patient | United Kingdom | Attended |
| 14 | Female | Patient | United Kingdom | Attended |
| 15 | Female | Patient | United Kingdom | Attended |
| 16 | Female | Patient | United States of America | Attended |
| 17 | Female | Charity/Support Group Representative | United Kingdom | Attended |

Supplementary appendix 11 - COSMIC: Intervention consensus meeting voting results.

| **No.** | **Question** | **Vote type** | **Round 1** | | | **Round 2** | | |
| --- | --- | --- | --- | --- | --- | --- | --- | --- |
|  |  |  | **G1 vote result** | **G2 vote result** | **Result** | **G1 vote result** | **G2 vote result** | **Result** |
| **1** | Do you agree that the two included outcomes “disease progression” and “response to treatment” should be merged into one outcome called “response to treatment”? | Binary (yes) | 100% (n=6) | 100% (n=11) | MERGE |  |  |  |
| **2** | How important is it that the outcome “tumor size” is included in the COS? | Likert (7-9) | 40% (n=5) | 42% (n=10) | OUT |  |  |  |
| **3** | How important is it that the outcome “adverse events during surgery” is included in the COS? | Likert (7-9) | 0% (n=6) | 18% (n=11) | OUT |  |  |  |
| **4** | How important is it that the outcome “social functioning” is included in the COS? | Likert (7-9) | 60% (n=5) | 70% (n=10) | OUT |  |  |  |
| **5** | How important is it that the outcome “role functioning” is included in the COS? | Likert (7-9) | 40% (n=5) | 80% (n=10) | UNDECIDED | 60% (n=5) | 70% (n=10) | OUT |
| **6** | How important is it that the outcome “emotional functioning” is included in the COS?​ | Likert (7-9) | 80% (n=5) | 80% (n=10) | IN |  |  |  |
| **7** | How important is it that the outcome “seizure control” is included in the COS? | Likert (7-9) | 100% (n=4) | 60% (n=10) | UNDECIDED | 100% (n=4) | 50% (n=10) | UNDECIDED* |
| **8** | How important is it that the outcome “need for further treatment” is included in the COS? | Likert (7-9) | 84% (n=6) | 100% (n=10) | IN |  |  |  |
| **9** | How important is it that the outcome “withdrawal from trial” is included in the COS? | Likert (7-9) | 17% (n=6) | 10% (n=10) | OUT |  |  |  |
| **10** | Do you agree that this should be the final COSMIC Intervention Core Outcome Set?​ | Likert (7-9) | 100% (n=5) | 100% (n=10) | ACCEPT |  |  |  |

Supplementary appendix 12 - COSMIC: Observation consensus meeting voting results.

| **No.** | **Question** | **Vote type** | **Round 1** | | | **Round 2** | | |
| --- | --- | --- | --- | --- | --- | --- | --- | --- |
|  |  |  | **G1 vote result** | **G2 vote result** | **Result** | **G1 vote result** | **G2 vote result** | **Result** |
| **1** | How important is it that the outcome “tumor size” is included in the COS? | Likert (7-9) | 76% (n=8) | 73% (n=6) | UNDECIDED | 75% (n=8) | 83% (n=6) | UNDECIDED* |
| **2** | How important is it that the outcome “growth-free survival” is included in the COS?​ | Likert (7-9) | 88% (n=9) | 83% (n=6) | IN |  |  |  |
| **3** | How important is it that the outcome “social functioning” is included in the COS? | Likert (7-9) | 78% (n=8) | 83% (n=6) | UNDECIDED | 88% (n=9) | 83% (n=6) | IN |
| **4** | How important is it that the outcome “role functioning” is included in the COS? | Likert (7-9) | 63% (n=8) | 100% (n=6) | UNDECIDED | 100% (n=9) | 100% (n=6) | IN |
| **5** | How important is it that the outcome “emotional functioning” is included in the COS?​ | Likert (7-9) | 63% (n=8) | 83% (n=6) | UNDECIDED | 100% (n=9) | 100% (n=6) | IN |
| **6** | How important is it that the outcome “neurocognitive functioning” is included in the COS? | Likert (7-9) | 76% (n=8) | 83% (n=6) | UNDECIDED | 100% (n=9) | 83% (n=6) | IN |
| **7** | Do you agree that this should be the final COSMIC Observation Core Outcome Set?​ | Likert (7-9) | 100% (n=9) | 100% (n=6) | ACCEPT |  |  |  |
